# Opportunities for groundwater microbial electro‐remediation

**DOI:** 10.1111/1751-7915.12866

**Published:** 2017-10-06

**Authors:** Narcís Pous, Maria Dolors Balaguer, Jesús Colprim, Sebastià Puig

**Affiliations:** ^1^ Laboratory of Chemical and Environmental Engineering (LEQUiA) Institute of the Environment University of Girona Campus Montilivi, Carrer Maria Aurèlia Capmany, 69 E‐17003 Girona Spain

## Abstract

Groundwater pollution is a serious worldwide concern. Aromatic compounds, chlorinated hydrocarbons, metals and nutrients among others can be widely found in different aquifers all over the world. However, there is a lack of sustainable technologies able to treat these kinds of compounds. Microbial electro‐remediation, by the means of microbial electrochemical technologies (MET), can become a promising alternative in the near future. MET can be applied for groundwater treatment *in situ* or *ex situ*, as well as for monitoring the chemical state or the microbiological activity. This document reviews the current knowledge achieved on microbial electro‐remediation of groundwater and its applications.

## Opportunities for microbial electrochemical technologies in groundwater treatment

Groundwater is one of the main sources of drinking water all over the world. However, its usage as drinking water is threatened by the presence of different pollutants that have reached the aquifer due to anthropogenic or geologic sources (Katsoyiannis *et al*., 2007; Bohlke *et al*., 2009; Van Halem *et al*., 2009; Sprague *et al*., 2011). The pollutants can be accumulated in the aquifer by the lack of a suitable electron donor/acceptor. But they need to be removed because of a further usage of drinking water or by means of environmental sustainability. The most used strategies are based on pollutant separation (membrane technologies or ion exchange) or external addition of chemicals for abiotic or biologic catalysis [e.g. organic matter for treating nitrates (McAdam and Judd, 2006) or oxygen/nitrate for treating hydrocarbons (Bamforth and Singleton, 2005)]. However, these technologies present some drawbacks. On the one hand, separation‐based technologies have a high energy cost and they concentrate the pollutant into a waste brine of difficult disposal (Twomey *et al*., 2010). On the other hand, the application of traditional remediating strategies that requires external chemical addition *in situ* or *ex situ* is limited by (i) undesired side reactions, (ii) poor chemical distribution (*in situ* strategies) and (iii) the addition of some chemicals can have collateral damages (e.g. organic matter addition can generate sludge that needs to be removed). Therefore, new sustainable strategies can have a golden opportunity on groundwater bioremediation if they have (i) low cost; (ii) no/low chemical consumption; and (iii) non‐invasive and selective electron donor/acceptor dosing. These characteristics can be met in microbial electrochemical technologies (MET), which are an emerging technology platform where microbiology meets electrochemistry (Schröder *et al*., 2015). In this technological approach, electroactive bacteria are able to use a solid electrode as electron donor or electron acceptor (Rabaey *et al*., 2009). The electrode is the alternative to oxygen/nitrate as electron acceptor (Bamforth and Singleton, 2005; Oremland and Stolz, 2005), or organic matter/hydrogen as electron donor (McAdam and Judd, 2006; Karanasios *et al*., 2010). Depending on the pollutant and groundwater's characteristics, a MET system can be operated as a microbial fuel cell (MFC) or as a microbial electrolysis cell (MEC) (Schröder *et al*., 2015). MFC is an autonomous device from where energy can be extracted, while a MEC is a device where energy is supplied to allow/enhance a bioelectrochemical process.

**Figure 1:**
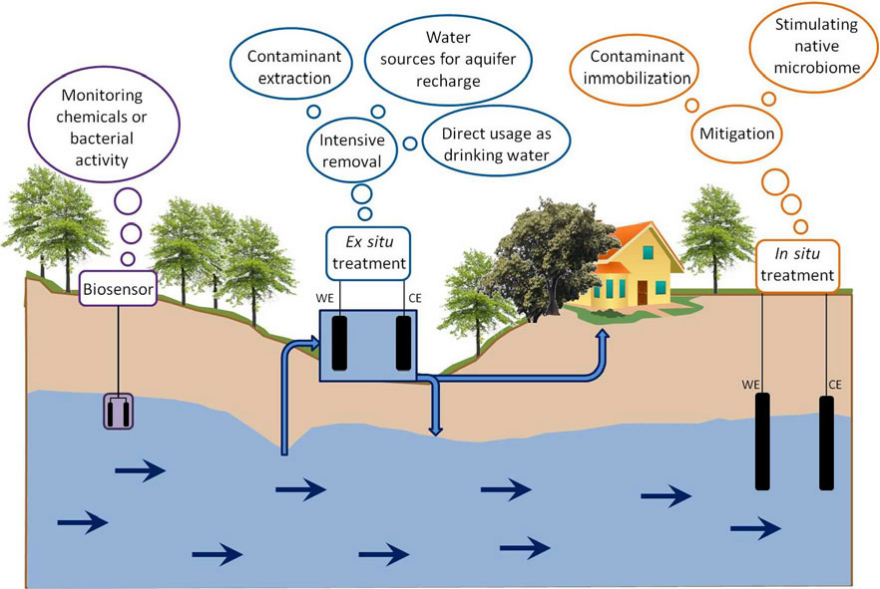
Framework of opportunities for microbial electrochemical technologies in groundwater.

Different commercial opportunities can be found for microbial electro‐remediation of contaminated groundwater (Fig. 1). The most studied application is the *ex situ* treatment. Through this strategy, groundwater has to be pumped to the treatment plant (either a permanent/*off‐site* or a movable/*on‐site* plant), where an intensive treatment is applied for a fast contaminant removal. The faster the treatment is, the smaller the plant volume is needed (with the corresponding decrease in the capital cost). The treated water can be either used for human purposes (i.e. drinking water) or re‐injected into the aquifer (i.e. to avoid salinity intrusion or to control the phreatic level). However, these *ex situ* strategies might not be recommended for some applications. In some scenarios, an *in situ* MET that allows the treatment and immobilization of the contaminant in the subsurface might be more suitable. For example, in aquifers with geochemical U(VI) solubilization (Williams *et al*., 2013), its *in situ* conversion into an insoluble form U(IV) and consequent immobilization in the subsoil might be preferred. For an *in situ* MET implementation, a less intensive treatment can be applied. Electrodes can be directly introduced in the aquifer to stimulate the native microorganisms and, in consequence, to accelerate the aquifer bioremediation (Gregory and Lovley, 2005). An approach can be followed similar to already available technologies like electrokinetic remediation (Acar *et al*., 1995) or vitrification (Mulligan *et al*., 2001).

Last but not least, small MET systems can also be used as biosensors to monitor the microbial activity in the aquifer (Williams *et al*., 2010; Wardman *et al*., 2014) or to evaluate its chemical state (Feng *et al*., 2013; Webster *et al*., 2014; Velasquez‐Orta *et al*., 2017).

Considering these different MET configurations, the treatment of different groundwater pollutants has been investigated using a solid electrode either as electron sink [e.g. for oxidation of aromatic hydrocarbons (Zhang *et al*., 2010; Friman *et al*., 2013) or dissolved metals (Pous *et al*., 2015a)] or as electron donor (e.g. for reduction of nitrates (Pous *et al*., 2013; Zhang and Angelidaki, 2013), metals (Gregory and Lovley, 2005) or chlorinated hydrocarbons (Aulenta *et al*., 2007). This review explores the MET platform for groundwater bioremediation.

## Organic contaminants

### Aromatic compounds

**Figure 2:**
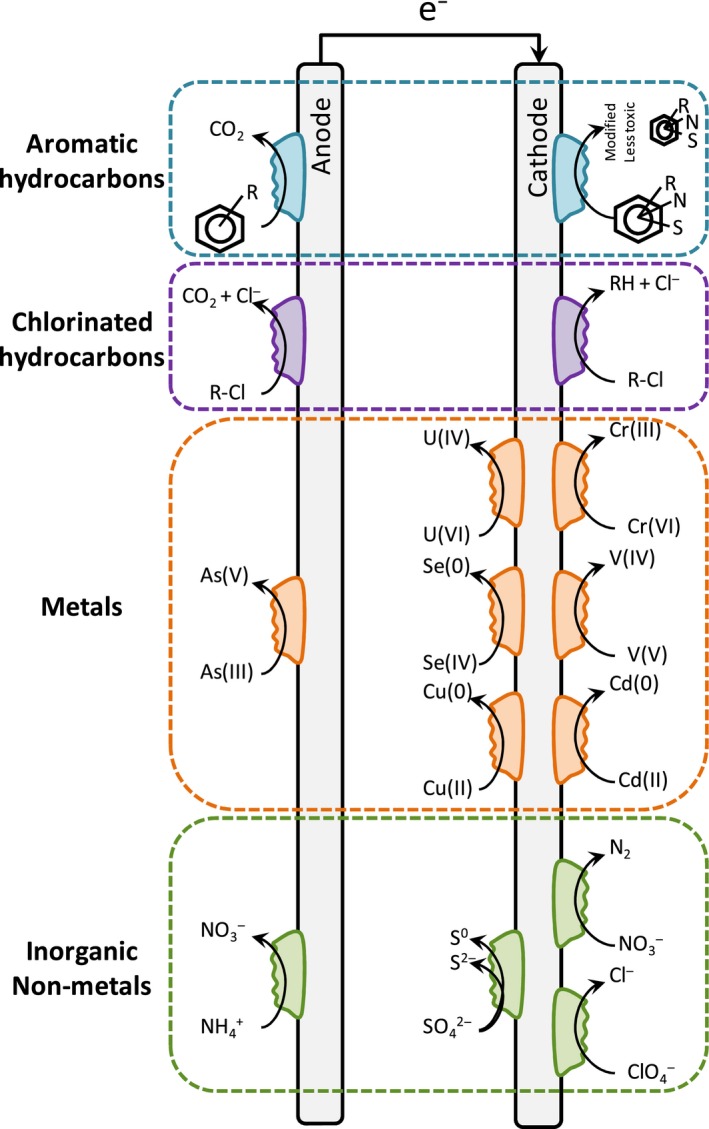
Summary of electrochemical reactions for the different pollutants treated in groundwater.

The presence of aromatic compounds in groundwater is mainly attributed to anthropogenic contamination, mostly derived from petrochemical activities (Turney and Goerlitz, 1990; Teuten *et al*., 2009). With a lack of electron acceptors, these substances can remain in the environment for a long time. Although the presence of aromatic hydrocarbons is usually found at low concentrations [μg·l^−1^ level (Rakoczy *et al*., 2013)], they are already toxic at these levels. For example, the guideline value for nitrobenzene in drinking water is 17 μg l^−1^ in the United States (Environmental Protection Agency (EPA), 2004). Therefore, a highly specific and effective treatment for this kind of compounds is needed, which can be difficult to achieve by conventional biologic treatments. The versatility of MET in terms of operational mode [microbial fuel cell (MFC), microbial electrolysis cell with 2‐ or 3‐electrode configuration (2‐MEC, 3‐MEC)], working electrode potential or active microbiome allows a plethora of aromatic contaminants to be treated (Table 1 and Fig. 2). Both MFC and MEC have proved to be an effective strategy to catalyse either polycyclic [e.g. phenantrene or naphthalene (Zhang *et al*., 2010; Yan and Reible, 2015)] or monocyclic aromatic compounds [e.g. benzene, phenol or nitrobenzene (Mu *et al*., 2009; Zhang *et al*., 2010; Friman *et al*., 2013; Rakoczy *et al*., 2013)]. Although electricity can be harvested from an MFC, it requires of stable and reliable counterelectrode reaction, which implies extramaintenance and surveillance. Thus, MEC operation might be preferred for bioremediation, as it allows focusing on aromatic compounds removal. Moreover, if an electrode control strategy is chosen (3‐MEC), a better control of the reaction and the remediation rates could be reached.

**Table 1:** Summary of organic pollutants treated in groundwater using microbial electro‐ remediation

Pollutant	Reaction	Placement	Operational mode	WE potential (mV vs. SHE)	Dominant associated microbiome	References
Polycyclic (PAHs)						
Phenantrene	Phenantrene → CO_2_	*In‐situ*	2‐MEC	+100	–	(Yan and Reible, 2015)
Naphthalene	Naphthalene → CO_2_	*Ex‐situ*	3‐MEC	+497	*Geobacter metallireducens*	(Zhang *et al*., 2010)
Azo dye orange 7 (AO7)	Azo dye → Sulfanilic acid	*Ex‐situ*	3‐MEC	−400/−450	*Cloacibacillus*	(Yun *et al*., 2017)
Dibenzothiophene	–	*Ex‐situ*	MFC	–	–	(Rodrigo *et al*., 2014)
Aromatic compounds						
Monocyclic						
Nitrobenzene	Nitrobenzene →Aniline	*Ex‐situ*	MFC	−495	–	(Mu *et al*., 2009)
			2‐MEC	–	Enterococcus	(Wang *et al*., 2011)
			3‐MEC	−400/−450	Cloacibacillus	(Yun *et al*., 2017)
Benzene	Benzene → CO_2_	*Ex‐situ*	3‐MEC	+497	*Geobacter metallireducens*	(Zhang *et al*., 2010)
			MFC	–	δ‐*proteobacteria*	(Rakoczy *et al*., 2013)
				–	*Chlorobiaceae, Rhodocyclaceae, Comamonadaceae*	(Wei *et al*., 2015a)
				–	–	(Wei *et al*., 2015b)
		In‐situ	MFC	–	–	(Chang *et al*., 2016)
Toluene	Toluene → CO_2_	*Ex‐situ*	3‐MEC	+497	*Geobacter metallireducens*	(Zhang *et al*., 2010)
Phenol	Phenol → CO_2_	*Ex‐situ*	3‐MEC	+322	*Cupriavidis basilensis*	(Friman *et al*., 2013)
			MFC	–	–	(Hedbavna *et al*., 2016)
Atrazine	Atrazine → CO_2_ + NH_3_	*Ex‐situ*	3‐MEC	+797	–	(Domínguez‐Garay *et al*., 2017)
Chlorinated hydrocarbons						
Tetrachloroethene (PCE)	PCE → cis‐DCE	*Ex‐situ*	3‐MEC	−300	*Geobacter lovleyi*	(Strycharz *et al*., 2008)
	PCE → Ethene	*Ex‐situ*	3‐MEC	−500	*Acinetobacter* sp., *Rhodopseudomonas* sp., *Pseudomonas aeruginosa*,* Enterobacter* sp.	(Yu *et al*., 2016)
Trichloroethene (TCE)	TCE → Ethene+ Cl^−^	*Ex‐situ*	3‐MEC	−500	–	(Aulenta *et al*., 2007)
				−653	–	(Aulenta *et al*., 2008)
				−550	*Dehaloccoides* sp.	(Aulenta *et al*., 2010)
				−450	–	(Aulenta *et al*., 2011)
				−250/−450	–	(Verdini *et al*., 2015)
				−650	–	(Lai *et al*., 2017)
cis‐Dichloroethene (cis‐DCE)	cis‐DCE → Ethene+ Cl^−^	*Ex‐situ*	3‐MEC	−550	*Dehaloccoides* sp.	(Aulenta *et al*., 2010)
	cis‐DCE → CO_2_+Cl^−^	*Ex‐situ*	3‐MEC	+1500	*Bacillus* sp.	(Aulenta *et al*., 2013)
				+1200	–	(Lai *et al*., 2017)
1,2‐Dichloroethane (1,2‐DCE)	1,2‐DCE → Ethene + Cl^−^	*Ex‐situ*	3‐MEC	−300	*Dehalococcoides*	(Leitão *et al*., 2015)
					*Dehalococcoides mccartyi*	(Leitão *et al*., 2016)
Clorophenol (CP)	2‐CP → Phenol	*Ex‐situ*	3‐MEC	−300	*Anaeromyxobacter dehalogenans*	(Strycharz *et al*., 2010)

WE accounts for Working Electrode; MFC indicates Microbial Fuel Cell; 2‐MEC indicates a Microbial Electrolysis Cell with a 2‐electrodes configuration and 3‐MEC accounts for a Microbial Electrolysis Cell with a 3‐electrodes configuration.

Like in other MET applications, this field of research became wider after it was found that *Geobacter* was able to oxidize aromatic compounds using an electrode as electron acceptor (Zhang *et al*., 2010). The ability of *Geobacter* sp. on dealing with aromatics can accelerate MET application in this field, as it is a well‐known and well‐studied model electroactive genus (Lovley *et al*., 2011). In fact, *Geobacter* species had already been detected in aquifers contaminated with aromatic compounds (Rooney‐Varga *et al*., 1999), thus suggesting their capacity to anaerobically oxidize aromatic hydrocarbons. Some years later, anaerobic benzene oxidation by *Geobacter* was successfully proven (Zhang *et al*., 2012). And even the genes for anaerobic benzene oxidation were identified for *Geobacter metallireducens* (Zhang *et al*., 2014). Thus, it was not surprising that one of the first MET experiences on aromatics removal evaluated the ability of *Geobacter metallireducens* to oxidize toluene, benzene or naphthalene to carbon dioxide using a graphite electrode as electron sink (Zhang *et al*., 2010). Nevertheless, oxidation of anaerobic aromatic hydrocarbons using MET is not an easy task. Only one pure microorganism not belonging to *Geobacter* genus has been reported to be able to oxidize phenol using an electrode as electron acceptor, *Cupriavidis basilensis* (Friman *et al*., 2013). The study of pure cultures is important for their understanding, but for real groundwater bioremediation applications, the usage of pure cultures might not be feasible. Then, the usage of mixed cultures gains interest. One of the most successful examples is the treatment of benzene. Benzene remediation has been successfully applied for either *ex situ* (Rakoczy *et al*., 2013; Wei *et al*., 2015a,2015b) or *in situ* experiences using mixed communities (Chang *et al*., 2016). An interesting finding was described by Rakoczy *et al*. (2013). The authors proved the simultaneous oxidation of sulfide and benzene in an anode mostly dominated by δ‐proteobacteria (31%). Isotopic analyses revealed that small amounts of oxygen might be required to activate the benzene oxidation in their system (Rakoczy *et al*., 2013). Thus, in real practical applications for aromatic hydrocarbon treatment, a positive coexistence of different microbial metabolisms is expected to happen.

The complexity of aromatics can increase with the presence of N‐ or S‐functional groups, leading to the need for developing different strategies for their treatment. One example of functionalized aromatics treatment in MET is nitrobenzene degradation. In the ideal case, nitrobenzene would be converted into CO_2_ and ${\text{NH}}_{4}^{-}$. However, nitrobenzene complexity makes this task hard, and its solely reduction into aniline can be already seen as a success (Mu *et al*., 2009; Wang *et al*., 2011; Yun *et al*., 2017). In fact, nitrobenzene reduction to aniline already reduces the water toxicity. Following a similar strategy, toxicity reduction instead of full oxidation, METs have been used for azo dye orange 7 reduction into sulfanilic acid (Yun *et al*., 2017) and the toxicity of waters containing dibenzothiophene or atrazine has also been decreased (Rodrigo *et al*., 2014; Domínguez‐Garay *et al*., 2016). Atrazine is an interesting example, as it has been successfully mineralized (Domínguez‐Garay *et al*., 2017). This example shows the potential of METs over the treatment of complex aromatic compounds.

In conclusion, a big window of opportunities can be opened for microbial electro‐remediation of aromatic hydrocarbons, as METs are capable to treat not only homoaromatic hydrocarbons, but also those containing N‐ or S‐functional groups or heteroaromatic hydrocarbons. Nevertheless, there are still relevant challenges to be addressed. As a general overview of microbial electro‐remediation, there is a lack of experiences at pilot‐scale level, which is also occurring in the field of aromatic compounds removal. In this case, as aromatics contamination is mostly derived from petrochemical activities (Turney and Goerlitz, 1990; Teuten *et al*., 2009), the most appropriate strategy would be *in situ* bioremediation, but field testing is still scarce (Daghio *et al*., 2017). This lack of experience is relevant for aromatics bioremediation, as more hurdles are expected to be found when moving to the field. For example, in a real petrochemical spill, there are several polyaromatic hydrocarbons species, some of which might not be bioavailable for bacteria due to its high hydrophobicity, and some others might also be toxic (Bamforth and Singleton, 2005). Nevertheless, laboratory testing is still needed to find the catalytic routes. Toluene, benzene or naphthalene has been already successfully converted into carbon dioxide (Zhang *et al*., 2010), but when treating more complex compounds such as nitrobenzene, azo dye or dibenzothiophene, the conversion to carbon dioxide could not be reached.

### Chlorinated hydrocarbons

Chlorinated hydrocarbons can be found in groundwater at ppb level due to solvent spills that have leaked into the aquifer (Squillace *et al*., 2002; Moran *et al*., 2007). Chlorinated hydrocarbons have been conventionally removed from groundwater by means of separation technologies (i.e. ion exchange, reverse osmosis or nanofiltration) (Altalyan *et al*., 2016) or through permeable reactive barriers (Obiri‐Nyarko *et al*., 2014). But there is a biologic alternative to deal with these compounds: reductive dechlorination (Holliger and Schraa, 1994; Holliger *et al*., 1998). In the ideal scenario, it allows turning the chlorinated hydrocarbons into ethene and chloride. Following this approach, the removal of chlorinated compounds using MET platform has been widely investigated by operating the system as a MEC (Aulenta *et al*., 2008; Strycharz *et al*., 2008). Bioelectrochemical dechlorination of some aromatic hydrocarbons, like chlorophenol (Strycharz *et al*., 2010; Wen *et al*., 2013), has also been reported. However, most of the studies have been focused on the removal of chlorinated aliphatic hydrocarbons (CAHs), the occurrence of which is high in groundwater.

Tetrachloroethene/perchloroethene (PCE) reduction using a polarized cathode as electron donor has been demonstrated by either mixed cultures (Yu *et al*., 2016) or a pure culture (*Geobacter lovleyi*) (Strycharz *et al*., 2008). The main objective is to reduce PCE into ethene (Chambon *et al*., 2013). However, PCE is reduced through a sequence of reactions where trichloroethene (TCE), cis‐dichloroethene (cis‐DCE) and vinyl chloride (VC) are stable intermediates that can be accumulated (Chambon *et al*., 2013). When using *Geobacter lovleyi* at a poised cathode potential of −300 mV versus standard hydrogen electrode (SHE), PCE was reduced at a maximum rate of around 25 μmol day^−1^, which was similar to the values observed when using acetate as electron donor (Strycharz *et al*., 2008). However, PCE was only converted into cis‐DCE, which is still a toxic compound and needs further degradation. Positively, when using a mixed culture at −500 mV versus SHE, PCE could be finally degraded into ethene in batch mode (Yu *et al*., 2016). However, a minimum of 50% of initial PCE was accumulated as vinyl chloride, indicating that further process optimization is needed.

The most studied chlorinated aliphatic hydrocarbon using MET is trichloroethene (TCE) (Aulenta *et al*., 2007, 2008, 2010, 2011; Verdini *et al*., 2015; Lai *et al*., 2017). From the initial proof‐of‐concept (Aulenta *et al*., 2007), research has evolved towards the understanding of the whole process [electron transfer mechanism (Aulenta *et al*., 2007, 2010), cis‐DCE role as intermediate (Aulenta *et al*., 2010, 2013; Lai *et al*., 2015) or electron competitors such as methane generation (Aulenta *et al*., 2008, 2011) and nitrate/sulfate presence (Lai *et al*., 2015)]. Process optimization through cathode potential, mass transport or continuous‐flow operation has also been evaluated (Aulenta *et al*., 2011; Verdini *et al*., 2015; Lai *et al*., 2017), and it has allowed to increase the bioelectrochemical dechlorination rates from 14.2–22.4 μeq l^−1^ day^−1^ (Aulenta *et al*., 2010) to 121.8 μeq l^−1^ day^−1^ (Lai *et al*., 2017) in the last years. These rates are similar to values obtained in conventional reductive dechlorination (Shukla *et al*., 2014), which highlights the competitiveness of bioelectrochemical reductive dechlorination. However, despite ethene is the desired product of reductive dechlorination, VC has been commonly observed as the main final product (Aulenta *et al*., 2007, 2008, 2010, 2011). In order to solve this issue, an interesting approach where TCE is reduced to VC in the biocathode and VC is further aerobically oxidized to carbon dioxide in the anode has been successfully implemented and demonstrated (Lai *et al*., 2017).

The list of chlorinated aliphatic compounds treated in MET can be further extended to the successful treatment of 1,2‐dichloroethane (1,2‐DCA) (Leitão *et al*., 2015, 2016, 2017). Initially, the 1,2‐DCA conversion to ethene was evaluated at different cathode potentials from −300 to −900 mV versus SHE using a *Dehalococcoides*‐enriched microbial culture. The authors observed 1,2‐DCA conversion to ethene at −300 mV versus SHE, a potential at which it was deduced that direct electron uptake was the mechanism driving this process (Leitão *et al*., 2015). The work was further extended by investigating the effect of supplementing an external mediator [Anthraquinone‐2,6‐disulfonate (AQDS)] in a biocathode polarized at −300 mV versus SHE (Leitão *et al*., 2016). Through AQDS addition, the 1,2‐DCA dechlorination rate increased from 20 μmol l^−1^ day^−1^ in the first work (Leitão *et al*., 2015) to 65 μmol l^−1^ day^−1^ in the last one (Leitão *et al*., 2016). AQDS could even be immobilized on the electrode surface for an easier application (Leitão *et al*., 2017).

In conclusion, the experience on bioelectrochemical reductive dechlorination is already broad in MET field. In the recent years, a positive evolution took place that allowed increasing the removal rates up to values similar to conventional reductive dechlorination and a better understanding of the underlying fundamentals of bioelectrochemical dechlorination was obtained (i.e. thermodynamics or the reductive pathway). Although important challenges still need to be addressed for becoming a market product, such as more studies at pilot‐scale level or a higher specificity to ethene as final product, microbial electro‐remediation is a promising approach for treating chlorinated hydrocarbons in groundwater.

## Inorganic contaminants

### Metallic compounds

Metals can be present in groundwater mainly because of the aquifer's geochemistry, but also due to leakages from industrial contamination. METs have been used as a technological approach to deal with different metals such as hexavalent uranium (Gregory and Lovley, 2005), hexavalent chromium (Huang *et al*., 2010), arsenite (Pous *et al*., 2015a) or selenite (Catal *et al*., 2009) (Table 2 and Fig. 2). In these cases, the objective is to change the metal oxidation state to one that presents lower toxicity and/or lower solubility. Different strategies can be explored depending on the metal that needs to be treated. In geologic‐associated contamination [such as U(VI), As(III), V(V) or Se(IV)], *in situ* microbial electro‐remediation might be the best strategy with the aim to immobilize the chemical species in their natural habitat. While in anthropogenic contamination [such as Cr(VI), Cd(II) or Cu(II)], the *ex situ* operation can be more appropriate to decontaminate the aquifer or, in the case of copper, to further recover it (Ter Heijne *et al*., 2010).

**Table 2:** Summary of inorganic pollutants treated in groundwater using microbial electro‐remediation

Pollutant	Reaction	Placement	Operational mode	WE potential (mV vs. SHE)	Dominant associated microbiome	References
Metallic						
U(VI)	U(VI) → U(IV)	*Ex‐situ*	3‐MEC	−303	*Geobacter sulfurreducens Desulfotomaculum, Nitrosoccoccus*	(Gregory and Lovley, 2005)
		In‐situ	3‐MEC	−303		
As(III)	As(lll) → As(V)	*Ex‐situ*	3‐MEC	+497	δ, γ‐*proteobacteria*	(Pous *et al*., 2015a)
				+500	*Achromobacter* sp., *Ensifer* sp., *Sinorhizobium* sp.	(Nguyen *et al*., 2016d)
					*Klebsiella*	(Nguyen *et al*., 2017)
Se(IV)	Se(IV) → Se(0)	*Ex‐situ*	MFC	–	–	(Catal *et al*., 2009)
			3‐MEC	−300	*Cronobacter*	(Nguyen *et al*., 2016c)
Cr(VI)	Cr(VI) → Cr(III)	*Ex‐situ*	MFC	–	–	(Huang *et al*., 2010)
					*Shewanella* sp.	(Hsu *et al*., 2012)
					*Shewanella oneidensis*	(Xafenias *et al*., 2013)
					γ‐*proteobacteria*	(Wu *et al*., 2015)
					*Shewanella oneidensis*	(Xafenias *et al*., 2015)
					–	(Song *et al*., 2016)
			2‐MEC	−303	*Proteobacteria*	(Huang *et al*., 2015)
			3‐MEC		*Shewanella oneidensis*	(Xafenias *et al*., 2013)
Cu(II)	Cu(II) → Cu(0)	*Ex‐situ*	2‐MEC	–	*Proteobacteria*	(Huang *et al*., 2015)
			MFC	–	*Stenotrophomonas maltiphilia, Citrobacter* sp., *Pseudomonas aeruginosa, Stenotrophomonas* sp.	(Shen *et al*., 2017)
Cd(II)	Cd(II) → Cd(0)	*Ex‐situ*	2‐MEC	–	*Proteobacteria*	(Huang *et al*., 2015)
Non‐metallic						
${\;\text{NO}}_{3}^{-}$	${\;\text{NO}}_{3}^{-}$ → N_2_	*Ex‐situ*	2‐MEC	–	–	(Sakakibara and Kuroda, 1993)
					–	(Feleke *et al*., 1998)
					–	(Park *et al*., 2005)
					α, β, γ‐*proteobacteria, Flavobacteria*	(Park *et al*., 2006)
					–	(Tong *et al*., 2013)
					–	(Kondaveeti and Min, 2013)
					*Proteobacteria*	(Kondaveeti *et al*., 2014)
					–	(Huang *et al*., 2013)
					*Nitratireductor* sp., *Shinella* sp., *Aeromonas* sp., *Pseudomonas* sp., *Curtobacterium* sp., *Dyella* sp.	(Nguyen *et al*., 2015)
			3‐MEC	−303	*Geobacter* sp.	(Gregory *et al*., 2004)
					*Geobacter metallireducens*	
				−123	–	(Pous *et al*., 2015a,2015b,2015c)
				−700	*Shinella* sp., *Alicycliphilus* sp.	(Nguyen *et al*., 2016a)
			MFC	–	–	(Pous *et al*., 2013)
		In‐situ	2‐MEC	–	–	(Tong and He, 2013)
			3‐MEC	−700	*Thiobacillus* sp., *Paracoccus* sp.	(Nguyen *et al*., 2016b)
${\text{ClO}}_{4}^{-}$	${\text{ClO}}_{4}^{-}$ → Cl^‐^	*Ex‐situ*	3‐MEC	−303	*Dechloromonas*,* Azospira*	(Thrash *et al*., 2007)
					–	(Shea *et al*., 2008)
			MFC	–	β‐*proteobacteria, Bacteroidetes*	(Butler *et al*., 2010)
					*Bacteroidetes, Firmicutes*, γ‐*proteobacteria*	(Mieseler *et al*., 2013)
			2‐MEC	–	*Aureibacter* sp., *Fulvivirga* sp., *Thermotalea* sp., *Thauera* sp.	(Wang *et al*., 2014)
${\text{SO}}_{4}^{2-}$	${\text{SO}}_{4}^{2-}$ → S^2−^	*Ex‐situ*	2‐MEC	−260	–	(Coma *et al*., 2013)
			3‐MEC	−900	*Methanobacterium, Desulfovibrio*	(Pozo *et al*., 2015)
				−1100	*Methanobacteriales*	(Pozo *et al*., 2016)
	${\text{SO}}_{4}^{2-}$ → S^0^	*Ex‐situ*	3‐MEC	−800	*Desulfovibrio* sp., *Sulfuricurvum* sp.	(Blázquez *et al*., 2016)
S^2−^	S^2−^ → ${\text{SO}}_{4}^{2-}$	*Ex‐situ*	MFC	–	δ‐*proteobacteria*	(Rakoczy *et al*., 2013)
			MFC	–	*Alcaligenes* sp., *Paracoccus* sp.	(Rabaey *et al*., 2006)
			3‐MEC	−100		

WE accounts for Working Electrode; MFC indicates Microbial Fuel Cell; 2‐MEC indicates a Microbial Electrolysis Cell with a 2‐electrodes configuration and 3‐MEC accounts for a Microbial Electrolysis Cell with a 3‐electrodes configuration.

One of the most studied applications is the microbial electro‐remediation of uranium‐contaminated sites (Gregory and Lovley, 2005). In these sites, uranium is present in form of U(VI) and the most desirable strategy for its bioremediation is the *in situ* conversion of U(VI) to U(IV), which is relatively insoluble and allows uranium immobilization in the aquifer (Gavrilescu *et al*., 2009). One of the most common strategies to promote uranium immobilization is to spike acetate or ethanol into the aquifer to stimulate native microbial U(VI) reduction (Gavrilescu *et al*., 2009). The interesting finding for MET applications was that *Geobacter* genus had been abundantly detected and enriched in sites where uranium bioremediation was implemented (Anderson *et al*., 2003; Shelobolina *et al*., 2008; Holmes *et al*., 2015). Bioremediation of U(VI) using MET instead of dosing acetate could decrease the ecological impact of the treatment as well as their cost, as it would only require the implementation of electrodes to stimulate bacterial activity. For this reason, the bioelectrochemical reduction of U(VI) using *Geobacter* has been proved in controlled laboratory experiments, as well as in real contaminated aquifers (*in situ* experiences) (Gregory and Lovley, 2005). The results obtained were promising, as 87% of uranium was recovered on the electrode surface (Gregory and Lovley, 2005). Moreover, bioelectrochemical U(VI) reduction represented a breaking point for the MET field. Until that moment, MET research had been focused on developing systems that relied on microbes able to deliver electrons to an electrode (microbial bioanodes). But the finding that the well‐known *Geobacter* was also able to get electrons from an electrode to perform bioelectrochemical reduction of U(VI), fumarate or nitrate opened a new field of research: microbial biocathodes (Gregory *et al*., 2004; Gregory and Lovley, 2005). Although the understanding of microbial electron transfer fundamentals in bioanodes is abundant, the knowledge for biocathodes is still scarce (Rosenbaum *et al*., 2011). For this reason, investigations over how *Geobacter* is able to get electrons from an electrode can be seen as a lighthouse for biocathodes in general. For example, the finding that *Geobacter sulfurreducens* requires outer‐surface c‐type cytochromes, but not conductive pili (microbial nanowires), for the reduction of U(VI) is a relevant contribution to the understanding of microbial reduction of soluble extracellular electron acceptors (Orellana *et al*., 2013). Moreover, the *Geobacter* versatility can also be used to hypothesize future pollutants to be evaluated using MET‐based bioremediation. For example, as *Geobacter* is also able to reduce the soluble V(V) to the more insoluble V(IV), MET could also become an alternative process for bioremediating **vanadium**‐contaminated sites (Ortiz‐Bernad *et al*., 2004). However, until now, only one experience of biocathodic V(V) reduction has been reported so far, getting a removal efficiency of 76.8% (Zhang *et al*., 2015).

MET is also contributing on the bioremediation of one of the most harmful and abundant metallic contaminants, arsenic, which is found in groundwater as arsenite [As(III)]. Its chemistry is different from the two metals discussed above, uranium and vanadium, where the highest oxidation state (U(VI) and V(V), respectively), were mobile, and thus, a reduction was needed for immobilization. In the case of arsenic, As(III) is highly mobile, while As(V) (arsenate) is more insoluble. Thus, the purpose is to use a bioanode able to oxidize arsenite to arsenate using a solid electrode as electron acceptor. The first study on arsenite oxidation using MET did not rely on arsenite‐oxidizing microorganisms. It was focused on coupling a MFC with zero valent iron to produce H_2_O_2_, which was further used to oxidize As(III) to As(V) (Xue *et al*., 2013). In 2014, Webster *et al*. (2014) engineered *Shewanella oneidensis* to develop an arsenite‐specific biosensor (Webster *et al*., 2014). One year later, the biologic arsenite oxidation using an electrode as electron acceptor was evaluated and proved (Pous *et al*., 2015a). A biofilm predominantly covered by γ‐ and δ‐proteobacteria was able to perform the As(III) conversion at a poised anode potential of +497 mV versus SHE. From there on, the arsenite bioanode oxidation has been further investigated. The As(III) oxidation performance has been improved, and a maximum As(III) oxidation rate of 29.6 mgAs l^−1^ day^−1^ has been achieved (Nguyen *et al*., 2016d). Moreover, it has been obtained additional knowledge about the microbial ecology responsible of microbial As(III) electro‐remediation, and arsenite oxidation has been successfully coupled to cathodic nitrate reduction (Nguyen *et al*., 2016d, 2017).

Microorganisms able to catalyse arsenic oxidation are usually considered together with selenium players (Stolz *et al*., 2006). However, a different approach for dealing with Se, which is commonly found as selenite [Se(IV)], has been tested in METs. In this case, selenite was successfully reduced to elemental selenium in microbial biocathodes, which allowed its immobilization (Catal *et al*., 2009; Nguyen *et al*., 2016c). Moreover, the finding that the well‐known electroactive *Shewanella oneidensis* MR‐1 has the ability to convert Se(IV) into Se(0) opens the door for more investigations on selenium‐contaminated groundwater treatment (Li *et al*., 2014).

*Shewanella* sp. has also been associated to chromium electro‐remediation (Hsu *et al*., 2012; Xafenias *et al*., 2013, 2015). Chromium is commonly used in different industries, and it can finally be released in their effluent streams as Cr(VI). As a result, it can be found in some groundwater bodies. In microbial biocathodes, Cr(VI) can be converted into Cr(III) using either a MFC (Huang *et al*., 2010; Hsu *et al*., 2012; Xafenias *et al*., 2013, 2015; Wu *et al*., 2015; Song *et al*., 2016) or a MEC configuration (Xafenias *et al*., 2013; Huang *et al*., 2015). The basis of the process is to convert the soluble Cr(VI) into a less soluble form, Cr(III). However, chromium can precipitate on the *Shewanella* surface (Kim *et al*., 2014), which could be seen as a limiting factor at long‐term operation. Nevertheless, the ability of MET to convert and anchor Cr(VI) can allow effluent concentrations below 5 ppb, which is below the guideline values for drinking water (Hsu *et al*., 2012).

In conclusion, microbial electro‐remediation is a versatile technology that allows the treatment of different metal contaminants, and it can be applied *in situ* or *ex situ* depending on the contaminant.

## Non‐metallic inorganic contaminants – nutrients

The presence of inorganic non‐metallic contaminants can be found in different groundwater bodies. MET has been proposed as an alternative method for nitrates (Pous *et al*., 2013; Zhang and Angelidaki, 2013), ammonium (Wei *et al*., 2015a), sulfates (Coma *et al*., 2013; Pozo *et al*., 2016) and perchlorates (Butler *et al*., 2010) (Table 2 and Fig. 2). Nitrate (Menció *et al*., 2011; Sprague *et al*., 2011), ammonium (Mastrocicco *et al*., 2013; Scheiber *et al*., 2016) and perchlorate (Bohlke *et al*., 2009; Izbicki *et al*., 2015) are mainly found in groundwater due to anthropogenic activities. In contrast, sulfates can also be accumulated because of aquifer's geology (Burg *et al*., 2017) and seawater intrusion (Bottrell *et al*., 2008), but it poses a lower risk for human health (Liamleam and Annachhatre, 2007).

Nitrates are one of the most widespread contaminants threatening groundwater's usage as drinking water. It can be found in several regions around the world as the bad face of intensive agriculture and livestock production (Menció *et al*., 2011; Sprague *et al*., 2011). Separation‐based technologies, such as reverse osmosis, reverse electrodialysis and ion exchange have been used to deal with nitrates in groundwater. These technologies are effective on removing nitrate, but they are energy‐intensive and they produce waste brine concentrated with nitrates of difficult disposal (Twomey *et al*., 2010). For this reason, technologies based on converting nitrates (to dinitrogen gas preferably) are being investigated. They can be divided into two main groups: abiotic and biologic. The abiotic alternatives are mainly based on electrocatalysis or the usage of a chemical catalyser, such as zero valent iron (ZVI) (Duca and Koper, 2012; Fu *et al*., 2014). Besides they could become effective strategies for removing nitrate, their main challenge is the low reduction specificity to dinitrogen gas (N_2_) as end‐product. Nitrate is converted into ammonium in most of the cases, which requires a post‐treatment (Duca and Koper, 2012; Fu *et al*., 2014). On the contrary, biologic treatments rely on bacteria, which are considered to be low‐cost and self‐renewable catalysers. Bacteria are able to convert nitrate into dinitrogen gas through the denitrification process. Biologic nitrate removal in METs has been widely studied because of its possible application to wastewater treatment (Clauwaert *et al*., 2007; Virdis *et al*., 2010; Puig *et al*., 2011; Pous *et al*., 2015b; Vilajeliu‐Pons *et al*., 2015). Although bioelectrochemical dissimilatory nitrate reduction (i.e. nitrate conversion to ammonium) has been described (Sander *et al*., 2015), nitrate removal in METs naturally follows the conventional denitrifying pathway in most of the cases (Clauwaert *et al*., 2007; Virdis *et al*., 2008). Nitrates are reduced to dinitrogen gas in the cathode compartment. However, literature regarding the treatment of nitrate‐polluted groundwater using MET is not abundant. The difference between treating nitrate in wastewater or groundwater using MET is relevant, as it has been demonstrated that the low conductivity of groundwater (≤ 1 mS cm^−1^) limits the MET performance (Puig *et al*., 2012). Thus, groundwater treatment is expected to have higher restrictions compared to wastewaters with higher conductivities and buffer capacities.

In the first studies regarding microbial electro‐remediation of nitrate, the mechanism was based on electrochemical water splitting to provide hydrogen to hydrogenotrophic denitrifiers (Sakakibara and Kuroda, 1993; Prosnansky *et al*., 2002). This process was considered an alternative to conventional hydrogenotrophic denitrification (Karanasios *et al*., 2010), in which hydrogen gas is directly supplied to a biological reactor. But this process is mass transfer limited due to the low solubility of hydrogen [1.6 mg l^−1^ at 20 °C (Soares, 2000)]. Sakakibara and Kuroda (1993) demonstrated that the complete reduction of nitrate to dinitrogen gas could be accomplished by applying different currents from 0 to 40 mA, which lead to increase the denitrification rate up to 0.15 mmol h^−1^. Although the authors stated that denitrification was mediated by H_2_ (produced *in situ* by electrochemical water splitting), it cannot be excluded that denitrification using the electrode as electron donor was taking place simultaneously. Besides the fact that the *in situ* electrochemical production of hydrogen for nitrate reduction was effective [nitrate removal rates up to 394 mgN l^−1^ day^−1^ (Prosnansky *et al*., 2002)], it implied a certain lack of process control. The hydrogen generated in the cathode may or may not be used for nitrate reduction. Hence, lower columbic efficiency can be expected for this type of configuration.

In 2004, Gregory and co‐workers observed that autotrophic denitrifiers were able to use a poised cathode electrode (−500 mV versus Ag/AgCl, −303 mV versus SHE) as electron donor, getting an electrode predominantly covered by *Geobacter* sp. (Gregory *et al*., 2004). Electron uptake from an electrode to perform denitrification was also demonstrated in groundwater (Park *et al*., 2005). In this case, by applying 200 mA, a nitrate removal rate of 435 mgN l^−1^ h^−1^ (10440 mgN l^−1^ day^−1^) was achieved in batch mode (Park *et al*., 2005). In groundwater, the electrode was predominantly covered by α‐, β‐, γ‐proteobacteria and Flavobacteriia, which indicated that not only *Geobacter* sp. (Gregory *et al*., 2004) were capable to perform bioelectrochemical denitrification. From there on, the investigation of nitrate removal in groundwater has been focused on determining the best operational strategies to increase nitrate removal rates.

If a MFC strategy is chosen to treat nitrate‐polluted groundwater, organic matter needs to be dosed into the anode compartment. Despite organic matter is not directly added to groundwater (it is added in a different compartment), it implies an extra cost. Hence, to convince future stakeholders that a BES operated as a MFC is suitable for groundwater bioremediation (Pous *et al*., 2013; Zhang and Angelidaki, 2013), the denitrification rates should be objectively higher than those obtained in conventional heterotrophic denitrification systems. By now, the highest denitrification rate reported in a denitrifying MFC has been around 500 mgN l^−1^ day^−1^ treating either groundwater (Zhang and Angelidaki, 2013) or synthetic wastewater (Clauwaert *et al*., 2009). A conventional heterotrophic treatment of nitrate‐polluted groundwater as membrane bioreactors (MBR) can reach values up to 1700 mgN l^−1^ day^−1^ (Wasik *et al*., 2001).

MET can be a market alternative for treating nitrate‐contaminated groundwater if it moves towards the idea of developing a fully autotrophic treatment. In this sense, a MEC operation is preferred, where external energy can be used to directly empower the denitrifying activity (Sakakibara and Kuroda, 1993). The fully autotrophic nitrate removal in groundwater has been evaluated in both MEC 2‐electrode (Sakakibara and Kuroda, 1993; Feleke *et al*., 1998; Park *et al*., 2005, 2006; Huang *et al*., 2013; Kondaveeti and Min, 2013; Kondaveeti *et al*., 2014; Nguyen *et al*., 2015) or 3‐electrode arrangement (Pous *et al*., 2015c; Nguyen *et al*., 2016a,2016b). Except for the case of Park *et al*. (2005), who reported 435 mgN l^−1^ h^−1^ in a 2‐MEC, and Pous *et al*. (2017), who reported 849 mgN l^−1^ day^−1^ in a 3‐MEC, the other authors obtained nitrate removal rates below 200 mgN l^−1^ day^−1^. A lower capital cost is required for a MEC 2‐electrodes, as it only needs a conventional power supply (e.g. power supply 0–30 V, 0–3 A has a cost of around 150 €). But MEC 2‐electrodes have a risk of side reactions (i.e. hydrogen evolution). On the contrary, the capital cost is higher for a MEC 3‐electrodes because a potentiostat is needed (e.g. potentiostat 0–20 V, 0–1 A has a cost of around 5000 €). However, in MEC 3‐electrodes, the cathode potential is controlled, which gives a better control over the electrode reactions. Thus, with both presenting advantages and disadvantages, the decision of choosing one or another will depend on each real application case.

In order to deliver drinking water, the plethora of configurations to deal with nitrate in groundwater is usually thought as *ex situ* applications (intensive treatment). However, experiences on *in situ* microbial electro‐remediation have also been explored, giving promising results (Tong and He, 2013; Zhang and Angelidaki, 2013; Nguyen *et al*., 2016b).

Another less common, but sometimes present, nitrogen compound is ammonium**.** It is a contaminant that can be found in subsurface waters that have received industrial or petrochemical pollution (Voyevoda *et al*., 2012). In those spills where oxygen is at low concentrations, ammonium is not oxidized into nitrate at the surface neither during the percolation (Buss *et al*., 2004). The main strategy to treat ammonium using METs is based on oxidizing ammonium aerobically into nitrate, which is then reduced into dinitrogen gas in a denitrifying biocathode (Virdis *et al*., 2008, 2010; Vilajeliu‐Pons *et al*., 2015, 2017). This strategy has been used to treat ammonium from real contaminated groundwater with satisfactory results in terms of ammonium oxidation, but low efficiencies of nitrate removal (Wei *et al*., 2015a,2015b). Wei *et al*., 2015a observed a 100% ammonium oxidation (20 mgN l^−1^) but an insufficient nitrate removal in a 0.16‐l reactor. While Wei *et al*., 2015b reached an stable ammonium removal of 100% during an operation time of 200 days in a MET presenting a 26 l volume and operated at 15 days HRT, but again an insufficient nitrate removal was observed. Another strategy that is being developed for treating ammonium is the ammonium oxidation using the anode as the final electron acceptor (Zhan *et al*., 2012, 2014; Zhu *et al*., 2016), but still low ammonium oxidation rates have been obtained [around 60 mgN l^−1^ day^−1^ (Zhan *et al*., 2014)].

Perchlorate is an emerging pollutant in groundwater, which consumption can cause a depression of thyroid hormone formation (Greer *et al*., 2002). The biologic treatment of perchlorate is performed by perchlorate‐reducing bacteria, which are able to convert ${\text{ClO}}_{4}-$ into Cl^−^. Besides no literature is available on perchlorate treatment in real groundwater, electro‐remediation of perchlorate in organic matter‐free media has been already proved (Butler *et al*., 2010). Like other biocathode‐based processes, the investigation of ${\text{ClO}}_{4}-$ reduction has been evaluated in MFC and MEC modes. Butler *et al*. (2010) were able to obtain electrical current by perchlorate cathodic reduction at a maximum rate of 24 mg l^−1^ day^−1^ (Butler *et al*., 2010). Under MEC mode, the perchlorate reduction was also possible at poised cathode potential of −500 mV versus Ag/AgCl (−303 mV versus SHE) (3‐electrodes) (Thrash *et al*., 2007) or by supplying a fixed current (2‐MEC) (Wang *et al*., 2014). However, the way to enrich this kind of reactors is one of the critical steps for MET application. For this reason, different inoculation strategies have been tested, such as the enrichment perchlorate‐reducing bacteria fed with acetate (Mieseler *et al*., 2013) or the adaptation of a denitrifying MET to perform perchlorate reduction (Shea *et al*., 2008). Both of them showed promising results, which should encourage further research on perchlorate bioremediation using METs.

Sulfates occurrence in groundwater also presents interest for microbial electro‐remediation, despite its low risk for human health. Some subsurface waters can present sulfate concentrations above the guideline value, and it also represents a risk for the utility infrastructure because of its possible conversion into hydrogen sulfide, even at low concentrations. Because of its low reduction potential [E^0^ (${\text{SO}}_{4}^{2-}$/HS^−^) = 0.252 V versus SHE, E^0^ (${\text{SO}}_{4}^{2-}$ ^/^S^0^) = 0.357 V versus SHE (Rabaey *et al*., 2009)] compared to organic matter oxidation [E^0^ (CH_3_COO^−^/${\text{HCO}}_{3}^{-}$) = 0.187 V versus SHE (Logan *et al*., 2006)], the reduction of ${\text{SO}}_{4}^{2-}$ in the cathode of a MFC is not feasible (Coma *et al*., 2013). Hence, it is necessary to apply external power to reach relevant removal rates. For example, Coma *et al*. (2013) observed a sulfate removal rate of 2 g${\text{SO}}_{4}^{2-}$ m^−3^ day^−1^ when operating as MFC (0 V applied), but a removal of around 65 g${\text{SO}}_{4}^{2-}$ m^−3^ day^−1^ when operating as MEC and applying 0.7 V. Not only the achievement of sulfate removal rates is important, but it is also important to determine which reduction product has been produced. In order to remove the sulfates from water using MET, two strategies have been evaluated: (i) sulfate conversion to sulfide, which could be extracted by promoting its precipitation as metal sulfide (Su *et al*., 2012; Coma *et al*., 2013; Pozo *et al*., 2016); (ii) sulfate conversion into elemental sulfur, which would allow S recovery for further usage if a cheap strategy for extraction is developed (Blázquez *et al*., 2016; Chatterjee *et al*., 2017). Nevertheless, the highest importance of studying sulfates bioelectrocatalysis for groundwater application is its coexistences together with other contaminants that posses higher risks for human health [e.g. together with chlorinated hydrocarbons (Lai *et al*., 2015) or with nitrates (Nguyen *et al*., 2016a)]. Therefore, the importance of the understanding of microbial electro‐remediation of inorganic non‐metallic pollutants in groundwater relies not only on the capacity of MET to treat these contaminants, but also on the possible interferences that these common contaminants can provoke to the electro‐remediation of others.

## Hurdles and challenges for groundwater microbial electro‐remediation

The scarcity of nutrients is one of the main hurdles that microbial electro‐remediation of groundwater has to face. From a chemical‐specific sight, N'Guessan *et al*. (2010) investigated the effect of phosphate limitation in *Geobacter* sp. The authors demonstrated that *G. sulfurreducens* is able to reduce U(VI) at phosphate‐limiting conditions (0.217 mM phosphate) (N'Guessan *et al*., 2010). Thus, the electroactive microorganism *G. sulfurreducens* was not limited by low nutrient availability, which gives good perspectives for their survival when treating groundwater.

From a general perspective, a clear indication of the low availability of chemical species itself is the low conductivity of groundwater (≤ 1.6 mS cm^−1^). The low conductivity can have a negative impact on MET, it implies higher ohmic and transport losses (Logan *et al*., 2006). For example, in the case of MET‐based nitrate removal, the decrease in conductivity from 4.3 to 1.3 mS cm^−1^ implied a decrease of 44% on nitrate removed (from 13.5 to 7.5 mgN l^−1^) (Puig *et al*., 2012). Moreover, the low conductivity can also lead to pH gradients by promoting to acidic pHs in the anode and basification in cathode. pH shifts can directly harm the electroactive bacteria and their removal performance (Clauwaert *et al*., 2008; Fornero *et al*., 2010), and it can lead to additional problems for the specific application of groundwater treatment. Depending on the aquifer's geochemistry, groundwater can present a high concentration of calcium, magnesium and bicarbonate (i.e. hardness) (Briggs and Ficke, 1977). The reductive nature of cathodes, together with the low buffering capacity of groundwater, can promote basified zones on the electrode surface. This induces scaling with the consequent blockage of the cathode electrodes, which can end up in MET deactivation (Santini *et al*., 2016). Besides it could be seen as a new application for MET (water softening) (Gabrielli *et al*., 2006; Zeppenfeld, 2011), strategies for solving this issue must be explored.

Another challenge for MET treatment of groundwater is the presence of mixtures of different contaminants (Squillace *et al*., 2002). The study of electro‐remediation of co‐contaminants in MET is limited, and few examples, such as perchlorate/nitrate (Xie *et al*., 2014) or cis‐DCE/nitrate/sulfate (Lai *et al*., 2015), can be found.

The cocktail perchlorate/nitrate is of a high interest, as they both can occur simultaneously (Dasgupta *et al*., 2005). On the one hand, anthropogenic perchlorate contamination has been linked to ammonium perchlorate (a missile propellant) (Hogue, 2003) and to nitrate‐based fertilizers, which also contain perchlorate (Susarla *et al*., 1999; Urbansky *et al*., 2000). It is relevant the case of the Chilean nitrate, since its perchlorate content is about 0.05–0.2 wt % ${\;\text{ClO}}_{4}^{-}$ (Urbansky *et al*., 2001). On the other hand, perchlorate can be naturally produced by sea salt aerosol photolysis in the atmosphere. This process can also involve nitrogen oxides, which can end up with nitrate deposition (Dasgupta *et al*., 2005). Xie *et al*. (2014) evaluated the occurrence of both nitrate and perchlorate in a MET. The experiments were performed in a perchlorate‐reducing biocathode grown at a poised cathode potential of −252 mV versus SHE (−500 mV versus SCE). After testing the perchlorate removal (initial concentration of 0.70 mM${\;\text{ClO}}_{4}^{-}$) together with different nitrate concentrations (0–2.10 m${\;\text{MNO}}_{3}^{-}$), the authors observed lower perchlorate reductions when higher nitrate concentrations were present. In batch experiments, a perchlorate concentration of 0.70 mM was totally consumed in 4 days when spiked alone. Twelve days were needed for its removal when 0.07 mM of nitrate was added, and perchlorate reduction was totally suppressed when nitrate was added at 2.10 mM (Xie *et al*., 2014). This inhibition of perchlorate reduction in the presence of nitrate is not specific of bioelectrochemical perchlorate reduction, and it has also been observed when using organic carbon or hydrogen as electron donors (Zhao *et al*., 2011; Ricardo *et al*., 2012). The reduction potentials of nitrate and perchlorate are similar (E^0^ ${\text{NO}}_{3}^{-}$/N_2_ = 1.25 V; E^0^ ${\;\text{ClO}\;}_{4}^{-}$/Cl^−^ = 1.28 V), which make them electron competitors (Bardiya and Bae, 2011). In fact, most of the perchlorate‐reducing bacteria identified so far are also able to denitrify (Nozawa‐Inoue *et al*., 2011). However, nitrate consumption allows higher cell growth. In consequence, the perchlorate reduction starts only after nitrate is depressed in most of the cases described (Bardiya and Bae, 2011). Hence, the decrease in perchlorate reduction in the presence of nitrate is linked to a substrate preference over nitrate. Thus, the tendency of bacteria over denitrification should be taken into account when dealing with a perchlorate/nitrate cocktail, and strategies for allowing perchlorate reduction should be implemented.

On the removal of cis‐DCE, the presence of nitrate and sulfate can also be possible, as they are one of the most widespread contaminants. For this reason, Lai *et al*. (2015) investigated whether nitrate and sulfate presence could affect bioelectrochemical reductive dechlorination of cis‐DCE (Lai *et al*., 2015). They observed that the cathode potential had a key role on selecting the target pollutant. In the cathode potential range evaluated (−550/−750 mV versus SHE), nitrate reduction always took place. As cathode potential was lowered, sulfate reduction and methanogenesis increased their activity. Besides reductive dechlorination was not inhibited, the electricity consumption incremented due to crossed reactions at lower cathode potentials. In this case, reductive dechlorination contribution was < 1% of the electrons consumed. The effect of sulfate was also evaluated on bioelectrochemical nitrate reduction (Nguyen *et al*., 2016a). Nguyen and co‐workers compared the denitrifying activity with or without sulfate (50 mgS‐${\text{SO}}_{4}^{2-}$ l^−1^), and they observed that the presence of sulfate suppressed, somehow, the overall denitrifying activity. Not only the nitrate removal rate decreased but also nitrite was accumulated as undesired denitrification intermediate. Therefore, it would be welcomed a further understanding on chemical species that coexist with the target pollutant in groundwater.

## Outlook for the future of microbial electro‐remediation of groundwater

Microbial electro‐remediation represents a unique opportunity to develop a robust, resilient and sustainable technology in a circular economy context to deal with different contaminants that are already present in our groundwater bodies. A considerable development has been done in the last 20 years in this field. Contaminants of different chemical nature (e.g. polycyclic heteroaromatic hydrocarbons, nutrients or metals) have been successfully treated using microbial electrochemical technologies. The technology proved its flexibility, as it has been adapted for *ex situ* or *in situ* treatment applications depending on the target pollutant. Moreover, MET‐based knowledge can also be applied to develop biosensors for contaminant or microbial monitoring in groundwater. However, in order to keep paving the way to its future implementation, specific development might be required for each specific pollutant, as their characteristics require different operational strategies. Strategies to overcome the restricting characteristics of groundwater and to face problems like carbonate scaling or those related to cocktails of contaminants need to be investigated and implemented. Moreover, testing at pilot plant level is still scarce, which demands an increase in scaling‐up orientated research to avoid technological stagnation.

## Conflict of interest

None declared.
